# Cost-effectiveness of using amyloid positron emission tomography in individuals with mild cognitive impairment

**DOI:** 10.1186/s12962-021-00300-9

**Published:** 2021-08-14

**Authors:** Young-Sil Lee, HyunChul Youn, Hyun-Ghang Jeong, Tae-Jin Lee, Ji Won Han, Joon Hyuk Park, Ki Woong Kim

**Affiliations:** 1 Department of Public Health Science, Graduate School of Public Health, Seoul National University, 1, Gwanak-ro, Gwanak-gu, Seoul, 08826 Republic of Korea; 2 Department of Psychiatry, Soonchunhyang University Bucheon Hospital, 170 Jomaru-ro, Wonmi-gu, Bucheon, 14584 Republic of Korea; 3 Department of Psychiatry, Korea University Guro Hospital, Korea University College of Medicine, 148, Gurodong-Ro, Guro-gu, Seoul, 08308 Republic of Korea; 4 Korea University Research Institute of Mental Health, 148, Gurodong-Ro, Guro-gu, Seoul, 08308 Republic of Korea; 5 Institute of Health and Environment, Seoul National University, 1, Gwanak-ro, Gwanak-gu, Seoul, 08826 Republic of Korea; 6 Department of Neuropsychiatry, Seoul National University Bundang Hospital, 82 Gumi-ro 173beon-gil, Bundang-gu, Seongnam, 13620 Republic of Korea; 7 Department of Psychiatry, Jeju National University School of Medicine, Jeju National University Hospital, Aran 13 gil, Jeju-si, Jeju, 63241 Republic of Korea; 8 Department of Psychiatry, Seoul National University College of Medicine, 101, Daehak-ro, Jongno-gu, Seoul, 03080 Republic of Korea; 9 Department of Brain and Cognitive Science, Seoul National University College of Natural Sciences, 1, Gwanak-ro, Gwanak-gu, Seoul, 08826 Republic of Korea

**Keywords:** Alzheimer’s disease, Amyloid, Mild cognitive impairment, Cost-effectiveness, Early diagnosis

## Abstract

**Background:**

Amyloid positron emission tomography (PET) makes it possible to diagnose Alzheimer’s disease (AD) in its prodromal phase including mild cognitive impairment (MCI). This study evaluated the cost-effectiveness of including amyloid-PET for assessing individuals with MCI.

**Methods:**

The target population was 60-year-old patients who were diagnosed with MCI. We constructed a Markov model for the natural history of AD with the amyloid positivity (AP). Because amyloid-PET can detect the AP MCI state, AD detection can be made faster by reducing the follow-up interval for a high-risk group. The health outcomes were evaluated in quality-adjusted life years (QALYs) and the final results of cost-effectiveness analysis were presented in the form of the Incremental Cost-Effectiveness Ratio (ICER). To handle parameter uncertainties, one-way sensitivity analyses for various variables were performed.

**Results:**

Our model showed that amyloid-PET increased QALYs by 0.003 in individuals with MCI. The estimated additional costs for adopting amyloid-PET amounted to a total of 1250 USD per patient when compared with the cost when amyloid-PET is not adopted. The ICER was 3,71,545 USD per QALY. According to the sensitivity analyses, treatment effect of Donepezil and virtual intervention effect in MCI state were the most influential factors.

**Conclusions:**

In our model, using amyloid-PET at the MCI stage was not cost-effective. Future advances in management of cognitive impairment would enhance QALYs, and consequently improve cost-effectiveness.

## Key points

- Using amyloid-PET at the mild cognitive impairment (MCI) stage is not cost-effective based on incremental cost-effectiveness ratio and willingness-to-pay thresholds.
- According to sensitivity analyses, future advances in management of cognitive impairment could improve cost-effectiveness of amyloid-PET in individuals with MCI.

## Background

According to the worldwide trends of population aging, dementia has posed a huge impact on public health in almost all countries [1]. Thus, the World Health Organization and Alzheimer’s Disease International designated dementia as a global public health priority [2, 3]. Also in South Korea, there were an estimated 6,61,707 persons with dementia (about 9.8% of people aged 65 and over) [4]. Social costs, including direct and indirect costs, were estimated at 18,200 USD per year per patient with dementia [4]. In addition, public management costs for patients with dementia were estimated at a total 12,600 million USD [4].

Alzheimer’s disease (AD) is the most common cause of dementia [5] and it has a slowly progressing degenerative course [6]. Clinical experts have emphasized early diagnosis and treatment of AD [7] as this can enable more systematic management planning and education of patients/caregivers, and consequently lead to social cost-savings [7, 8].

Mild cognitive impairment (MCI) has been regarded as an intermediate state between normal aging and the earliest manifestations of dementia [9]. Individuals with MCI can exhibit varying results such as reversion to normal, sustained MCI, and conversion to dementia [10]. The annual rates of conversion from MCI to dementia have been known to be around 10–15% [10–12]. And the prevalence of MCI is known to be relatively high − 10% to 20% of individuals aged 65 or over [13–15]. Therefore, many previous researchers have focused on diagnosing prodromal AD in individuals with MCI.

Amyloid positron emission tomography (PET) imaging, using radioactive ligands that bind to amyloid plaques, makes it possible to diagnose AD in its prodromal phase including MCI [16, 17]. Many researchers have discussed the appropriate use of amyloid-PET and reported guidelines [18–21]. So far, routine use of amyloid-PET in individuals with MCI has not been generally recommended [18–21] because amyloid-PET only detects a brain histological state, not a clinical diagnosis and amyloid-lowering therapies have not been developed yet [20]. However, since amyloid-PET is helpful for early diagnosis and differential diagnosis of AD, the clinical use of amyloid-PET has been continuously considered [18–21]. In addition, many older adults want to know their disease status of cognitive disorder earlier [22]. In Korea, amyloid-PET is currently excluded from national insurance coverage, but discussions are ongoing on whether to include amyloid-PET in national insurance coverage in the future [23].

There are some previous studies that claim that amyloid-PET is cost-effective in predementia or similar states. Hornberger et al. evaluated the cost-effectiveness of adopting amyloid-PET in patients with early-stage or suspected AD in France and Spain and reported that this scanning cost-effectively increases quality-adjusted life years (QALYs) [8, 24]. Guo et al. also showed that adopting amyloid-PET in patients with predementia and dementia is cost-effective in the United States [25]. While these studies show the cost-effectiveness of adopting amyloid-PET, they did not selectively target patients with MCI. MCI patients are in an earlier stage of disease progression than those in these previous reports, and the results on cost effectiveness of amyloid-PET in MCI can be different from those in previous studies.

Therefore, economic analyses of the implementation of amyloid-PET in patients with MCI are needed. This study evaluated the cost-effectiveness of including amyloid-PET for assessing individuals with MCI. We believe that this may help determine healthcare policy, as well as dictate the indication for amyloid-PET.

## Methods

### Target population

The target population was 60-year-old patients who were diagnosed with MCI. MCI patients have identified cognitive impairment but there is uncertainty about whether they have developed AD. Therefore, there is a possibility of benefiting from additional interventions such as using amyloid-PET.

The age of patients was chosen based on previous studies and expert opinion that it is generally accepted to be earlier than the average age for the onset of AD [26, 27]. It was assumed that amyloid plaques accumulation was observable at this age.

### Comparator

We set the current practice as a comparator. In the current practice, amyloid-PET is not recommended for general MCI patients [18]. We compared the current practice with the implementation of amyloid-PET for all diagnosed MCI patients.

### Markov model

**Fig. 1:**
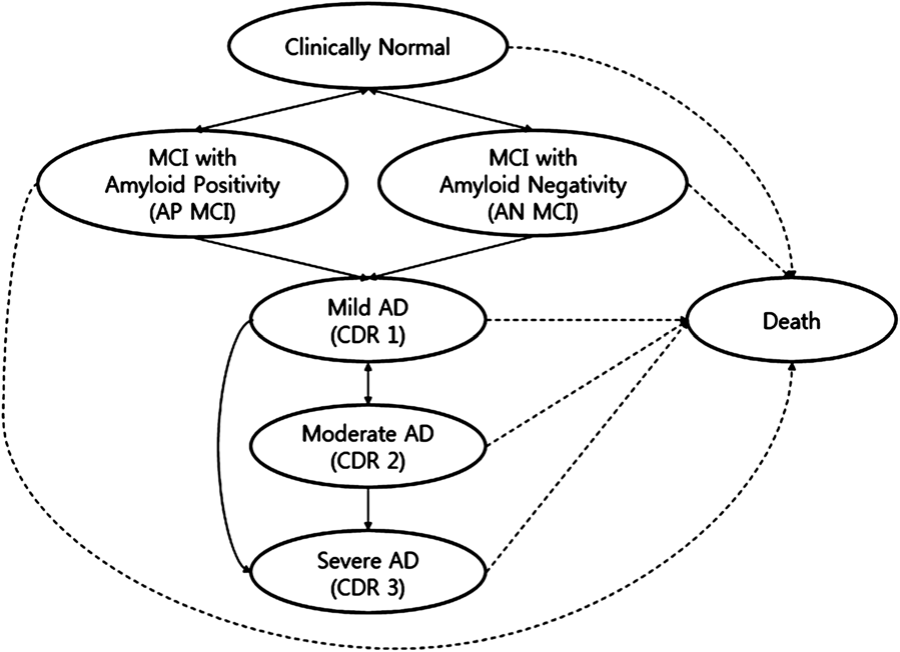
Schematic representation of the Markov model

We constructed a Markov model for the natural history of AD with the amyloid positivity (AP) and amyloid negativity (AN). Figure 1 is a schematic representation of this model.

### Model assumptions

#### Health states

The progression after AD incidence followed Yu et al.’s and Neumann et al.’s models [28, 29]. And the progression before AD incidence (from Clinically Normal to mild AD) followed experts’ opinion. It was assumed that non-AD dementia could occur but exited from the model to focus on the effects on costs and health outcomes caused by AD.

The initial condition in all population is MCI. Without amyloid-PET, MCI patients could not be classified as AP MCI or AN MCI. MCI is clinically reversible; however, once AD occurs, it becomes clinically irreversible. Moderate AD can convert to mild AD. AP MCI and AN MCI are not interchangeable and all states can lead to death.

#### Detection of AD

Moderate and severe AD are immediately detected because of the severity of their symptoms. However, in MCI and mild AD, the patients may not know their exact state. Thus, individuals in this category are only diagnosed at the time of the follow-up visit. As the follow-up interval becomes shorter, patients could know their condition more accurately. For a conservative estimation, mild AD was assumed to be detected 3 years after onset regardless of a follow-up.

#### The interval of follow-up

The interval of follow-up depends on an individual’s health state. We assumed that clinically normal persons did not get a follow-up, patients with MCI got a follow-up every year, and AD patients did every three months with reference to expert opinion. AP MCI patients were assumed to get a follow-up every 3 months after they were identified by amyloid-PET. AN MCI patients were assumed to get annual follow-up visits.

#### Treatment of AD

The treatment was assumed to start from the moment AD was detected. Therefore, if a patient had not been confirmed as having AD by a doctor, he or she would not be treated even if they actually had mild AD. At the MCI state, any type of treatment was not assumed. AD treatment was thought to slow down the progression to a more severe cognitive status.

#### Expected benefit of amyloid-PET

Because amyloid-PET can detect the AP MCI state, AD detection can be made faster by reducing the follow-up interval for a high-risk group. Early detection means early treatment thereby slowing the AD disease progression.

### Parameters

Table 1 shows the model parameters and values applied in the analyses. We used the parameters extracted from Koreans and then used overseas data if there were no appropriate Korean-specific data.

**Table 1:** Model parameters and values applied in the analyses

Parameters	Point estimates (range for sensitivity analysis)	References
Annual transition probabilities		
Clinically normal MCI	0.0341	KLOSCAD^a^
MCI clinically normal	0.0641	KLOSCAD
AN MCI mild AD	0.0211	KLOSCAD
MCI non-AD dementia	0.0017	KLOSCAD
Mild AD moderate AD	0.3220	[29]
Mild AD severe AD	0.0420	[29]
Moderate AD mild AD	0.0430	[29]
Moderate AD severe AD	0.3390	[29]
Excess mortality risk^b^		
Moderate AD	2.57	[29]
Severe AD	7.82	[29]
Prevalence of amyloid positivity in MCI	0.3910 (0.3617, 0.5556)^c^	Author^d^
Relative risk of AD conversion of AP MCI	7.95 (3.53, 15.20)^c^	SR
Diagnostic accuracy of amyloid-PET		
Sensitivity	0.92	[35]
Specificity	1	[35]
Quality of life weight		
Clinically normal (age ≥ 65)	0.874	KNHANES
MCI	0.80	[36]
Mild AD	0.43	[36]
Moderate AD	0.21	[36]
Severe AD	0.17	[36]
Medical cost per year ($)^e^		
Clinically normal	0	–
MCI	794	[36]
Mild AD	2113	[36]
Moderate AD	1478	[36]
Severe AD	1819	[36]
Non-medical cost per year ($)		
Clinically normal	0	–
MCI	2539	[36]
Mild AD	10,956	[36]
Moderate AD	11,796	[36]
Severe AD	14,273	[36]
Amyloid PET cost ($)	1041 (520, 1561)^f^	Author
Additional follow-up cost per 1 visit ($)	27 (14, 41)^f^	HIRA + [36]
Treatment effect	0.85 (0.70, 0.93)^g^	SR
Virtual intervention effect for AP MCI^h^	1 (0.95, 1)	Assumption
Start age	60 (60, 80)	–
Discount rate	0.05	–

*AD* Alzheimer’s disease, *AN* amyloid negative, *AP* amyloid positive, *HIRA* Korean Health Insurance Review & Assessment Service, *KNHANES* Korea National Health and Nutrition Examination Survey, *MCI* Mild Cognitive Impairment, *SR* Systematic Review result by authors

^a^Korean longitudinal study on cognitive aging and dementia. The values in the table are unpublished data. For a description of the KLOSCAD cohort, see Han et al.[30]

^b^Neumann et al. did not directly present excessive mortality risks [29]. Authors calculated it based on their annual probabilities

^c^The minimum and maximum value were obtained from Doraiswamy et al. [31], Ong et al. [32], Schreiber et al. [33], and Thurfjell et al.[34]

^d^The values derived from survey or clinical study performed with this economic evaluation study

^e^All costs were measured in 2017 and converted as follows: 1,130 KW to 1 USD

^f^The range of sensitivity analyses for cost items, ± 50% of the base-case value was set to the range

^g^The minimum and maximum value were obtained from individual studies included in the synthesis

^h^If the effect is 0.95, it means that the probability of moving from AP MCI state to mild AD state will be 0.95 times

### Transition probabilities

Annual transition probabilities between each health state were obtained from the Korean longitudinal study on cognitive aging and dementia (KLOSCAD) cohort and from Neumann et al. [29, 30]. When AD was detected and treatment initiated, the adjusted probabilities were applied.

### The excess mortality risks of AD

MCI and mild AD were assumed to have the same mortality rates as the rates seen in the clinically normal state, which were calculated based on age-specific life tables from the Korea National Statistical Office. But moderate and severe AD were assumed to have excess mortality risks, which were calculated based on Neumann et al.’s annual probability [29].

### The prevalence of AP MCI

When amyloid-PET was taken in 312 patients diagnosed with MCI, 122 (39.1%) patients were classified as AP MCI. The results were obtained from MCI patients who underwent amyloid-PET at Korea University Guro Hospital or Seoul National University Bundang Hospital. Please e-mail us for more details.

### Relative risk of AD conversion from AP MCI

Though the other probabilities in the model were obtained from cohort studies, there were no reliable values for AD incidence rate based on the amyloid positivity state. To solve this problem, we conducted a systematic literature review to obtain the relative risk of AD conversion from AP MCI when compared with the rate of conversion from AN MCI. The prospective studies that observed MCI patients, whose baseline amyloid positivity state was known over a certain period of time so as to observe the incidence of AD, were collected. Finally, four studies were selected [31–34] and the relative risk was calculated by dividing the rate of AP MCI converting to AD by the rate of AN MCI converting to AD. As a result, we found that AD occurs in AP MCI 7.95 times more than in AN MCI. We assumed that the incidence of AD in AN MCI was equal to the incidence of AD in general MCI.

### Diagnostic accuracy of amyloid-PET

The diagnostic accuracy of amyloid-PET in this study indicates its ability to distinguish between AP and AN. It is related to whether or not amyloid accumulation in the brain is higher than threshold only and is not related to the presence of dementia or MCI. We used the results obtained from an A16 phase trial, which was the only study that used autopsy to confirm amyloid accumulation in the brain [35].

### Health-related quality of life

Health-related quality of life was measured using the Korean version of the EQ-5D-3L in a survey (Seoul National University IRB No. 1707/003–021) linked to this study. Participating patients were stratified by severity and the quality of life weights were also calculated by severity [36]. Severity classification followed the Clinical Dementia Rating (CDR) [37].

### Costs

The costs of this study were derived from the healthcare system perspective. Direct medical costs, direct non-medical costs, time and travel costs for both patients and caregivers, care costs including caregivers’ time costs, and long-term care costs were included. Productivity costs were excluded.

All costs, except direct medical costs, were calculated from the survey described above [36]. The responses were averaged by CDR severity and adjusted as an annual cost. Direct medical costs were calculated by reviewing the hospital records and using only the costs associated with dementia.

Amyloid-PET testing costs and follow-up costs were calculated by multiplying the unit cost by the frequency of uses. There is no formal amyloid-PET unit cost because amyloid-PET is not reimbursed by National Health Insurance (NHI) in Korea. Therefore, the average cost of the three hospitals that participated in the study was applied. Follow-up cost is the sum of consultation fee, travel cost, and time cost. Consultation fee was taken from NHI and travel and time cost were calculated from the survey.

All costs were measured in 2017 and converted as follows: 1,130 KW to 1 USD, using the International Monetary Fund's official exchange rate [38].

### Treatment effects

We used synthesis-based estimates when applying treatment effects to AD patients. Donepezil was set as the standard of treatment drug and individual literature was selected in the Cochrane review published in 2018 [28, 39]. Only those studies with the Clinician’s Interview-Based Impression of Change scale (CIBIC-plus) or Clinical Global Impression of Improvement scale (CGIC) outcomes were selected to reflect the comprehensive improvement of AD symptoms. After extracting the necessary information from the selected studies, the relative risk (RR) was calculated using Review Manager 5.3 with the fixed effect model chosen based on a small number of studies and non-significance of heterogeneity test. The synthesized RR was 0.85 (95% CI: 0.80–0.90), implying that the probability of an AD patient progressing to an advanced state will be lower by 0.85 times when treated with Donepezil compared to placebo.

### Analysis

The analysis was initiated at age 60 and all subjects were analyzed until death. It might be better to set the time horizon as a lifetime to include all the costs and health outcomes caused by AD. The cycle length of the Markov model was set to 3 months to reflect the change in the follow-up interval and half-cycle correction was applied. We used TreeAge Pro 2019, R2 (TreeAge Software, Inc., Williamstown, MA).

The health outcomes of our study were evaluated in QALYs, which make it possible to reflect on the major disease characteristics of AD, i.e., the declining quality of life. Both costs and health outcomes were discounted at an annual rate of 5%, as recommended by Korean Health Insurance Review & Assessment Service (HIRA) guidelines. The final results of cost-effectiveness analysis were presented in the form of the Incremental Cost-Effectiveness Ratio (ICER).

### Sensitivity analyses

To handle parameter uncertainties, one-way sensitivity analyses for various variables were performed and presented in the form of tornadogram.

Base-case analysis assumed no treatment when a patient was in the MCI state, but for comparison with similar studies [8, 24, 25], we performed a sensitivity analysis assuming the existence of virtual interventions that could reduce the incidence of AD when in the AP MCI state. In such a case, patients identified as AP MCI via amyloid-PET would receive additional benefits.

The range of sensitivity analyses used minimum and maximum values if there were previous studies. For the cost items, ± 50% of the base-case value was set to the range. Exceptionally, the annual management cost, which is the sum of annual medical cost and non-medical cost, used input values of a previous study [25] to compare the result directly. The values of the previous study were used after adjusting for the inflation rate.

Written informed consent was obtained from all the participants and their legal guardian. The necessary ethical permissions were received from the Institutional Review Board at Korea University Guro Hospital (2016GR0003/2016GR0200), Jeju National University Hospital (2016–06-017), Seoul National University (1707/003–021), and Seoul National University Bundang Hospital (B-1608/360–007).

## Results

Table 2 shows the result of base-case analysis. In MCI patients, the cost was $60,037 without amyloid-PET and $61,287 with amyloid-PET, i.e., a $1,250 increased. QALYs were 8.757 and 8.760, respectively, showing an increase by 0.003 with amyloid-PET. The ICER was $371,545 per QALY.

**Table 2:** Cost-effectiveness of amyloid-PET for MCI patients

	Cost($)	ΔCost($)	QALYs	ΔQALYs	ICER
No amyloid-PET	60,037		8.757		
Do amyloid-PET	61,287	1250	8.760	0.003	3,71,545^a^

*QALYs* quality-adjusted life years, *ICER* Incremental Cost-Effectiveness Ratio

^a^This ICER is sensitive to small changes in the QALY difference caused by rounding off error

**Fig. 2:**
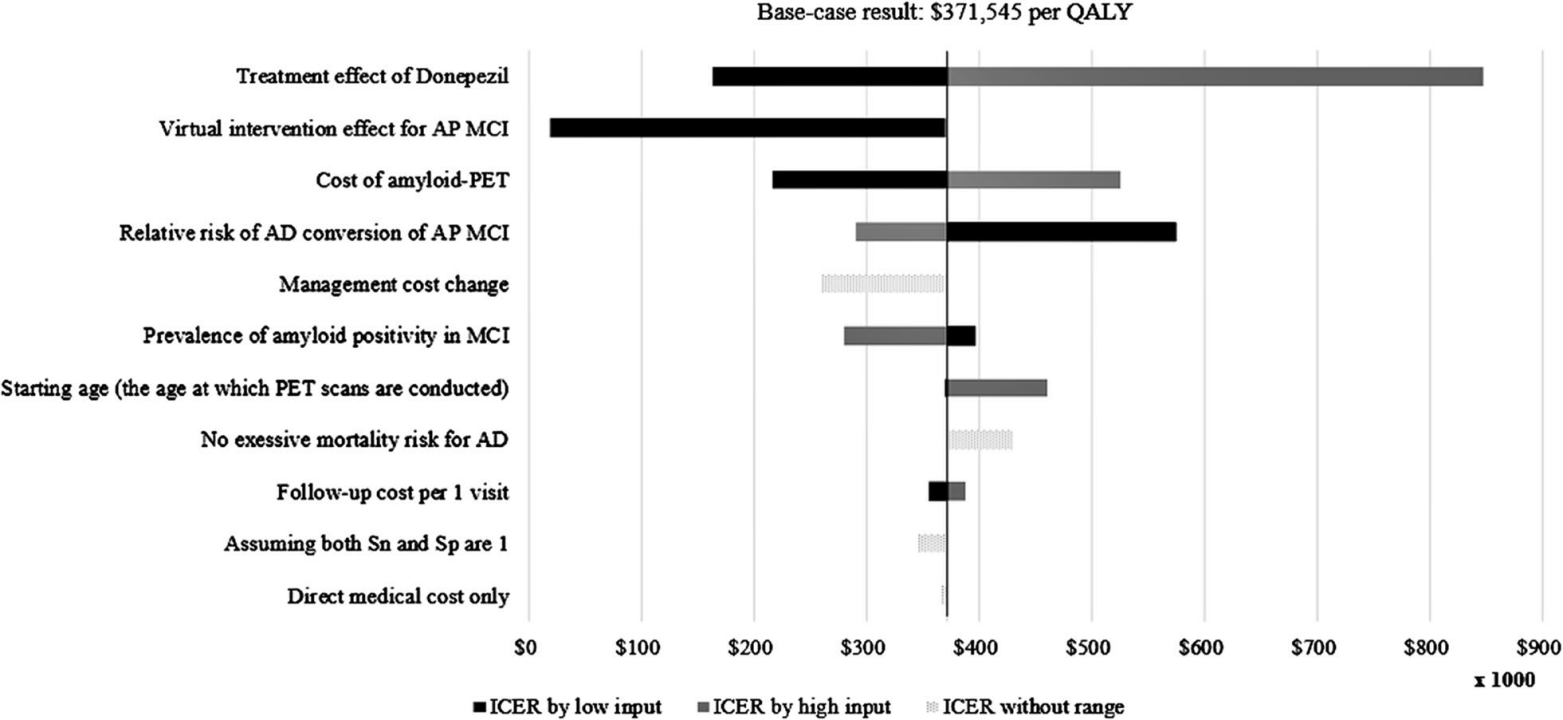
The tornadogram of one-way sensitivity analyses

Figure 2 shows the main results of one-way sensitivity analyses. Treatment effect of Donepezil and virtual intervention effect for AP MCI were the most influential factors. In particular, it seemed cost-effective (ICER $19,461 per QALY) when the lowest virtual intervention effect for AP MCI (0.95) was applied. In all other cases, ICERs were larger than $160,000 per QALY.

## Discussion

Our results showed that amyloid-PET increased QALYs by 0.003 in individuals with MCI. The estimated additional costs for adopting amyloid-PET in individuals with MCI amounted to a total of 1250 USD per patient when compared with the cost when amyloid-PET is not adopted. Consequently, the ICER of our model was 3,71,545 USD per QALY. The threshold for ICER varies in countries. 30,000 EUR (i.e., 35,000 USD), 40,000 EUR (i.e., 47,000 USD), and 50,000 USD per QALY were affordable ICER in Spain, France, and in the United States studies on cost-effectiveness of adopting amyloid-PET, respectively [8, 24, 25]. In South Korea, though there is no official threshold for ICER, willingness-to-pay per QALY was estimated at 27,000 USD in 2012 [40]. Even after considering inflation, the ICER of our model is considerably higher than the willingness-to-pay per QALY in South Korea.

As far as we know, three economic evaluation studies about 18F amyloid-PET have been published so far [8, 24, 25] and our result is the complete opposite of that of previous studies. We believe that this difference may be due to several different settings and assumptions.

First, the assumptions related to disease progression are different. Previously Spain, France, and United States studies assumed that cognitive functioning would only deteriorate into dementia from the MCI state if not treated [8, 24, 25]. This assumption can increase the benefits of amyloid-PET by rating the benefits of early detection highly. Unlike these studies, we assumed the possibility of reversion to clinically normal from the MCI state (0.0641). Some previous studies have reported that individuals with MCI could return to cognitively normal states. Koepsell et al. showed that 16% of subjects diagnosed with MCI reverted back to normal or near-normal cognition approximately one year later [41]. Malek-Ahmadi conducted a meta-analysis and found an overall reversion rate of approximately 24% [42]. Our assumption reflects these previous studies.

Second, assumptions about treatment are different. In our model, it was assumed that treatment could not be started immediately, even if brain amyloid accumulation was detected in the elderly with MCI. That is, we assumed that treatment for dementia began only when the progression to dementia was detected by reflecting the policy and guideline of NHI in South Korea [43]. Time of treatment initiation in previous models of Spain and France was assumed earlier than that of our model [8, 24]. Considering the common assumption that dementia medications have some effect in the course of cognitive decline, a delayed start of treatment in our model may have contributed in part to our high ICER of amyloid-PET.

In particular, Guo et al. assumed that virtual treatment during the predementia phase would reduce the risk of conversion by 50% among those patients with prodromal AD [25]. To date, the evidence for interventions to reduce the risk of conversion to dementia in predementia is very limited [44–47]. As a result of the sensitivity analysis using similar assumptions for the comparison with the previous studies, we found that the cost-effectiveness of amyloid-PET was greatly improved as in Guo et al. [25]. This implies that amyloid-PET could be a cost-effective alternative if interventions that would significantly reduce the risk of conversion from MCI to dementia were developed, but not cost-effective at the moment. In addition, previous studies have assumed that the treatments for AD have no effect on non-AD dementia. Actually, there are pieces of evidence that these are also effective in vascular dementia [48] or Parkinson’s disease dementia [49]. Thus, the above assumption may have exaggerated the benefits of amyloid-PET.

Third, Korea has a relatively low care costs for severe AD than other countries. In our study, the annual care cost of severe AD was 16,092 USD (medical cost of 1,819 USD + non-medical cost of 14,273 USD), which was approximately 3,000 USD different from mild AD (13,069 USD) or moderate AD (13,274 USD). Although the severity classification method and the cost estimation method were different, in Guo et al., the annual care cost was about 14,000 USD for mild state, 34,000 USD for moderate state, and 60,000 USD for severe state [25] and in the remaining two studies, it was assumed that the care time and cost increased rapidly with severity. The time to care for severe patient was more than twice the amount of time needed to care for mild patient [8, 24]. If the cost difference between the severities is large, the benefit from early detection becomes bigger. Our sensitivity analysis result was consistent with this. However, when we inserted the annual management cost of the previous study in the United States, the reduced ICER was still as high as 2,60,789 USD.

There are some limitations to our analysis. First, because amyloid-PET is a relatively recent technology, some data related to it are limited. For example, there were no reliable values for the AD incidence rate based on the state of amyloid positivity; therefore, we carried out a systematic literature review and sensitivity analysis. If there was a large-scale cohort study, it would be possible to obtain a robust value and also obtain values by age.

Second, we did not include reduction in the use of other tests caused by including amyloid-PET because we believe that such an analysis would be more reflective of real-life clinical practice. We believe that amyloid-PET is not yet a replacement of brain structural imaging, F-18 fluorodeoxyglucose-PET, and neuropsychological testing in real clinical practice. However, some studies have reported that amyloid-PET can be associated with reduction of other tests. Johnson et al. included reduction of other imaging and neuropsychological testing in anticipated impact of amyloid-PET [18]. Grundman et al. investigated potential impact of amyloid imaging in 229 patients with a history of cognitive decline and uncertain diagnosis, and reported that amyloid-PET reduced brain structural imaging by 24.4% and neuropsychological testing by 32.8% [50]. In this respect, our model may have underestimated the benefits of amyloid-PET.

Third, we eliminated non-AD dementia from our model. This setting may underestimate the benefit of amyloid-PET because amyloid-PET helps to more accurately distinguish types of dementia in patients with dementia. However, since our target population were MCI patients who had not yet developed dementia, and some of the treatments for AD patients are commonly used for patients with non-AD dementia [48, 49], the setting where amyloid-PET can more accurately diagnose the dementia type, leading to an effective treatment, poses a risk of overestimating the benefits of amyloid-PET. Further studies are needed to clarify the economic benefits of amyloid-PET populations including non-AD dementia patients.

Fourth, we implemented one-way sensitivity analyses to address parameter uncertainties, which failed to consider distributional concerns or correlation between parameters. A probabilistic sensitivity analysis would be preferred in future studies.

## Conclusion

This is the first study that has assessed the cost-effectiveness of adopting amyloid-PET in individuals with MCI in South Korea. According to our model, using amyloid-PET at the MCI stage is not cost-effective. Future advances in management of cognitive impairment could enhance QALYs, and consequently improve cost-effectiveness if amyloid-PET is added to manage individuals with MCI. We believe that our findings have provided more real-life evidence to the present and future healthcare policies with regards to the addition of amyloid-PET in the management of MCI.

## Data Availability

The datasets used and/or analyzed during the current study are available from the corresponding authors on reasonable request.

## Abbreviations

AD: Alzheimer’s disease
AN: Amyloid negativity
AP: Amyloid positivity
CDR: Clinical Dementia Rating
CGIC: Clinical Global Impression of Improvement scale
CIBIC-plus: Clinician’s Interview-Based Impression of Change scale
HIRA: Korean Health Insurance Review & Assessment Service
ICER: Incremental Cost-Effectiveness Ratio
KLOSCAD: Korean longitudinal study on cognitive aging and dementia
MCI: Mild cognitive impairment
NHI: National Health Insurance
PET: Positron emission tomography
QALYs: Quality-adjusted life years
RR: Risk ratio
